# Enhancing T Cell Chemotaxis and Infiltration in Glioblastoma

**DOI:** 10.3390/cancers13215367

**Published:** 2021-10-26

**Authors:** Kirit Singh, Kelly M. Hotchkiss, Kisha K. Patel, Daniel S. Wilkinson, Aditya A. Mohan, Sarah L. Cook, John H. Sampson

**Affiliations:** Duke Brain Tumor Immunotherapy Program, Department of Neurosurgery, Duke University Medical Center, Durham, NC 27710, USA; kelly.hotchkiss@duke.edu (K.M.H.); kisha.patel@duke.edu (K.K.P.); daniel.wilkinson@duke.edu (D.S.W.); aditya.mohan@duke.edu (A.A.M.); sarah.quackenbush@duke.edu (S.L.C.)

**Keywords:** immunotherapy, glioblastoma, blood–brain barrier, central nervous system, T cells, T lymphocytes

## Abstract

**Simple Summary:**

Immunotherapy in glioblastoma has so far failed to yield a survival benefit. This failure can be attributed to a paucity of immune cells at the tumor site which can be reinvigorated to kill tumor cells. Therefore, driving effector immune cells such as cytotoxic T lymphocytes to the tumor is a necessary pre-requisite of any effective immunotherapy approach. In this review, we will discuss therapeutic approaches possible for trafficking T cells from the periphery to travel through the blood–brain barrier and tissue of the brain to reach the tumor.

**Abstract:**

Glioblastoma is an immunologically ‘cold’ tumor, which are characterized by absent or minimal numbers of tumor-infiltrating lymphocytes (TILs). For those tumors that have been invaded by lymphocytes, they are profoundly exhausted and ineffective. While many immunotherapy approaches seek to reinvigorate immune cells at the tumor, this requires TILs to be present. Therefore, to unleash the full potential of immunotherapy in glioblastoma, the trafficking of lymphocytes to the tumor is highly desirable. However, the process of T cell recruitment into the central nervous system (CNS) is tightly regulated. Naïve T cells may undergo an initial licensing process to enter the migratory phenotype necessary to enter the CNS. T cells then must express appropriate integrins and selectin ligands to interact with transmembrane proteins at the blood–brain barrier (BBB). Finally, they must interact with antigen-presenting cells and undergo further licensing to enter the parenchyma. These T cells must then navigate the tumor microenvironment, which is rich in immunosuppressive factors. Altered tumoral metabolism also interferes with T cell motility. In this review, we will describe these processes and their mediators, along with potential therapeutic approaches to enhance trafficking. We also discuss safety considerations for such approaches as well as potential counteragents.

## 1. Introduction

Immune surveillance of the central nervous system (CNS) is essential for environmental homeostasis and pathogen clearance. Without immune surveillance, opportunistic infections in the CNS commonly develop [1]. However, the entry of immune cells into the CNS is tightly controlled by the blood–brain barrier (BBB) and the blood–cerebrospinal fluid (BCSF) barrier. These formidable barriers lack fenestrations, exhibit a low degree of pinocytosis, and are sealed together by a network of intracellular junctions [2,3]. While this close control is desirable in health to avoid runaway immune responses in the CNS, restricted immune cell entry severely hampers the effectiveness of immunotherapy in glioblastoma [4]. This is further complicated by the immunosuppressive tumor microenvironment (TME), which consists of endothelial cells, pericytes, fibroblasts, and regulatory immune cells [5]. The TME drives effector immune cell exhaustion, thereby shielding solid malignancies from immune attack [6]. While immune checkpoint inhibition (ICI) seeks to reverse this exhausted state and ‘release the brakes’ on regional T cells, it is notable that the evaluation of resected stage IV gliomas are either devoid or demonstrate limited numbers of tumor-infiltrating lymphocytes (TILs) [7,8]. This would suggest that ICI will struggle owing to the lack of targets to reinvigorate. Indeed, initial trials of ICI in glioblastoma have failed [9]. However, when ICI is combined with increased numbers of functional TILs in pre-clinical models, long-term survival can be achieved [10,11]. Therefore, we require therapeutic strategies that can both recruit effector cells to the tumor site and ensure they remain functional.

While the CNS does host several immune cell classes, including T cells, these immune cells are clustered away from the tumor-bearing parenchyma in regions such as the choroid plexus, the meninges (containing the subarachnoid and perivascular spaces), and the CSF [12,13,14,15,16,17]. The clinical implications of this clustering were recognized as long ago as 1923, where Murphy and Sturm confirmed Shirai’s initial finding that foreign tumors in the parenchyma could grow, but tumors implanted close to the ventricles (and thus the immune interfaces) were rejected [18]. Fortunately, immune responses in the CNS can be bolstered by an adaptive response originating from the periphery. Medawar demonstrated in 1948 that tumors implanted into brain tissue can be rejected following exposure to tumor antigens outside of the CNS [19]. Recruitment of peripheral T cells into the parenchyma also occurs in multiple sclerosis (MS) and its animal analogue experimental autoimmune encephalitis (EAE) [20].

Even though adaptive immune clearance of tumors is possible, glioblastoma possesses several mechanisms that suppress the recruitment and functioning of T cells. Glioblastoma expresses decreased levels of lymphangiogenesis-promoting factors such as VEGF-C, reducing potential routes for T cell ingress, while the highly immunosuppressive tumor microenvironment (TME) blunts the response of any lymphocytes that reach the tumor [21,22,23]. Therefore, in this review we will discuss the physiological processes that drive T cell trafficking from the periphery, tumoral infiltration, and potential therapeutic options for their enhancement. We will also discuss safety considerations, given the potential for T cell infiltration to drive inflammation and neurodegeneration in the CNS [24,25].

## 2. T Cell Trafficking from the Periphery to the Blood–Brain Barrier

The mechanism by which T cells leave the circulation and enter inflamed tissues is well characterized and has been reviewed in detail elsewhere [26,27,28]. In brief, the expression of selectins on endothelial cells results in the slowing and rolling of leukocytes. The leukocyte crawls along the endothelial layer, where stimulating chemokines trigger the activation of integrins, which ultimately result in the leukocyte being firmly captured [29,30,31]. Engagement of endothelial adhesion molecules by integrins results in immune cells being drawn to endothelial junctions, permitting their diapedesis (crossing) into the tissue [32]. T cell trafficking across the BBB involves a similar process of rolling, capture, and diapedesis [33]. However, certain aspects of this process differ from the periphery. In the resting state, the constitutive expression of selectin is largely absent in the CNS, with the exception of blood vessels in the sub arachnoid space [34,35]. T cell rolling on the BBB is instead driven by the cell surface integrin LFA-1, which binds to intercellular adhesion molecule-1 (ICAM-1) on the endothelium. These T cells are captured and cross via G protein-coupled receptor (GPCR) signaling [36].

In the pathological state, the release of inflammatory cytokines induces the expression of chemokines and adhesion molecules that recruit effector T cells to the CNS [37,38]. Transcription and expression of E- or P-selectins on the BBB adheres to P-selectin glycoprotein ligand-1 on CD8^+^ T cells, inducing their slowing on the endothelial surface [39]. Binding is again mediated by GPCR signaling, which activates the integrins LFA-1 and VLA-4 on the T cells that bind to ICAM-1 and VCAM-1, respectively [40]. Other factors at the BBB also interact with VLA-4, including transmembrane proteins described as junctional adhesional molecules (JAM). So far, JAM-B and JAML have been implicated in CD8 chemotaxis—blockade of JAM-B results in the reduced CNS infiltration of CD8^+^ T cells [41,42]. Atypical chemokine receptor-1 (ACKR1) also mediates trafficking in the inflammatory state, transporting pro-infiltrative chemokines to the luminal aspect of the BBB [43]. Interleukin (IL)-1 signaling in BBB endothelial cells is associated with upregulated expression of VCAM-1, ICAM-1, and ACKR1 and therefore may offer a potential strategy for enhancing T cell capture if delivered intra-tumorally [44]. Frewert et al. reported that intra-tumoral infusion of IL-1β or interferon-γ via convection enhanced delivery enhanced the number of CD4^+^ and CD8^+^ TILs in a rat glioma model [45]. This may therefore be a rational combinatorial approach alongside ICI.

It should be noted that the expression of these adhesion molecules is also influenced by perivascular stromal cells such as regional pericytes [46,47]. In health, these cells co-ordinate with endothelial cells to control both the development and permeability of the vasculature [48]. Pericytes also inhibit endocytosis by endothelial cells, limiting transcellular routes of migration [49,50]. Indeed, mice deficient in pericytes display significantly increased expression of VCAM-1 and ICAM-1 on the BBB endothelium, resulting in a mass influx of leukocytes [51]. In the tumor setting, overgrowth of pericytes derived from glioma stem cells (GSCs) results in the blockage of the entrance of therapeutic drugs such as temozolomide (TMZ) [52]. Pericyte coverage is inversely correlated with survival in patients with glioblastoma following chemotherapy [53]. Interestingly, selective targeting of these cells using ibrutinib was shown to enhance delivery of TMZ in orthotopic models of glioma by disrupting the blood–tumor barrier [53]. This may also have a double effect of interrupting the pericyte secretion of CCL5, which acts on CCR5 on glioblastoma cells, inducing resistance to TMZ by promoting DNA damage repair mechanisms [54]. However, pericytes can re-organize themselves to cover areas of deficient coverage and their function may be compensated for by other local cells such as astrocytes [55].

The CCL5–CCR5 axis is also associated with enhanced regulatory T cell recruitment [56]. CCR5 antagonists such as maraviroc (licensed for HIV-1) have been found to deplete regulatory T cells, which express CCR5/CXCR4 ratios differently to T effector cells [57,58]. Blockade of CCR5 has also been demonstrated to reduce the growth of orthotopically injected colon cancer cells by limiting cancer-associated fibroblast accumulation. Maraviroc has also been shown to reverse CCL5 resistance to TMZ and is also BBB penetrable with a favorable safety profile, making this an agent of significant interest as part of future combinatorial approaches [54,59,60]. CCR5 also binds CCL3 and CCL4 and the interaction between CCR5 and its ligands appears to have location-specific pro or anti-tumor effects [61]. For example, CCL4 can help to recruit cytolytic CCR5^+^ T cells in esophageal squamous cell carcinoma, but the CCL4–CCR5 interaction can enhance the invasion ability of glioblastoma in vitro [62,63].

To determine how best to drive T cells into the CNS, we need to identify the optimal pro-infiltrative phenotype of lymphocytes. A high expression of integrins and chemokine receptors as seen in autoimmune disease is likely beneficial for enhancing T cell chemotaxis in glioblastoma. When reviewing T cell phenotypes that predominate in autoimmune diseases, CNS-infiltrative lymphocytes are predictably dominated by effector memory T cells (CD62L_Lo_ and CCR7_Lo_) [64,65,66,67,68,69]. While sensitizing T cells in the periphery would be ideal, the high degree of heterogeneity in glioblastoma makes it impossible to identify a universal target [70]. A more optimal approach would be trafficking antigen-naïve T cells that can interact with antigen-presenting cells (APCs) that endogenously present tumor antigen. This strategy benefits from the fact that T cells do not require target antigen recognition before they are able to cross the BBB [71]. In glioblastoma, T cells primed in tumor-draining cervical lymph nodes strongly upregulated VLA-4, leading to preferential infiltration of the CNS [72]. Therefore, activating tumor-antigen naïve T cells to express VLA-4 will help to achieve this objective. Administration of IL-12 to mice bearing multiple tumor types appeared to enhance the induction of LFA-1 and VLA-4 and subsequently T cell migration, resulting in tumor regression [73]. However, this response differed across tumor types, with IL-12 resulting in largely CD4 migration in fibrosarcoma but pre-dominantly CD8 migration in ovarian cancer, and further work is required to determine its impact in glioblastoma [73].

While antigen specificity is not a pre-requisite of migration into the CNS, it is interesting to note that adoptively transferred tumor-naïve T cells appear to undergo a period of residence in the lungs, where their gene expression profile switches to a migratory phenotype [74]. Determining mediators of this ‘licensing’ process may therefore yield useful therapeutic targets of interest. Before entering the lungs, adoptively transferred T cells predominantly migrate towards homeostatic chemokines CCL19 and CCL21 (expressed in bronchus-associated lymphoid tissues). After transiting through the lungs, T cell homing shifts towards chemokine gradients associated with inflammation such as CXCL11 and CCL5 [74]. CXCL11 binds the chemokine receptor CXCR3 (expressed on effector T cells), and this binding can promote T cell infiltration into tumors [75]. Conversely, inhibition of CXCR3 binding results in reduced invasion of effector T cells [76].

**Figure 1:**
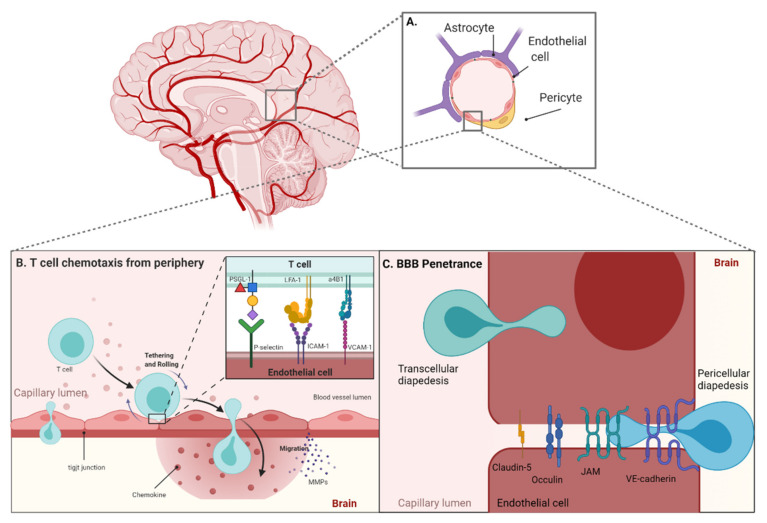
(**A**) The BBB consists of endothelial cells held together by tight junctions surrounded by pericytes and astrocytes. (**B**) T cell chemotaxis across the BBB is facilitated by expression of tethering molecules (P-selectin, ICAM-1, VCAM-1, etc.) on endothelial cell surfaces that bind to integrins on circulating T cells (LFA-1, α4β1, etc.) to slow and allow cells to roll across the membrane surface. (**C**) T cells can cross the endothelial cells either between cells (paracellular) through tight junctions or through individual cells (transcellular) to migrate into the brain. Produced using Biorender.

CXCR3 binds three ligands: CXCL9, CXCL10, and CXCL11. While CXCL11 binds CXCR3 with higher affinity, it also can induce receptor internalization and promote a regulatory T cell lineage [75]. Instead, CXCL10 may be a more suitable therapeutic approach, as CXCL10 induces moderate receptor internalization and still enhances T cell infiltration [77]. This was demonstrated in intracranial melanoma models, where the absence of CXCL10 was associated with decreased numbers of CD8^+^ TILs [78]. Although glioma does express CXCL10, this is accompanied by the expression of dipeptidylpeptidase (DPP)-4, which cleaves CXCL10 [79]. DPP-4 blockade has been shown to increase the numbers of TILs, but when considering therapeutic blockade, it must be noted that DPP-4 also inhibits glioma proliferation independently of its enzymatic activity [80,81]. An alternative approach to inducing expression of CXCL10 is the use of poly-ICLC, which has been found to significantly increase the frequency of TILs when combined with peptide vaccination against glioma [82]. An overview of peripheral T cell chemotaxis and BBB penetrance is shown in Figure 1.

## 3. Blood–Brain Barrier Specific Targets

Following the shift towards a pro-infiltrative phenotype, T cells must cross the BBB. Although glioblastoma is a disease state in which the BBB is disrupted, regions of the tumor are likely surrounded by intact portions of barrier [83]. These privileged regions may act as the site of regrowth, shielded from immunotherapeutic attack [8,83]. Such privileged regions correspond with the non-contrast enhancing infiltrating edge, which can form the site of recurrence following core resection [84]. Infiltrating glioma cells at the leading edge demonstrate upregulated fibroblast growth factor-mediated signaling that promotes tumorigenesis [85]. The changes in cellular phenotype at the leading edge are driven by histone deacetylase signaling from the tumor core [86]. This results in a permanent alteration at the border to a pro-infiltrative milieu of glioma-initiating cells, which does not reverse following resection of the core [86]. Immune cell populations differ at this interface zone also. Spatial single-cell RNA-Seq analysis performed by Darmanis et al. revealed that tumor associated macrophages (TAMs) dominated the core while brain-derived microglia dominated the peritumoral zone [87]. These both have key roles in T cell activity at the tumor site. Macrophages can express the cytokine TGF-β, which enhances glioblastoma cell growth, migration, and invasion and downregulates antitumor immunity [88,89]. Microglia in the peritumoral zone show an increased expression of ligands for T cell exhaustion-associated receptors such as PD1 and CTLA4 [87]. Microglia also express CCL2 and CCL5 which, as mentioned previously, enhances regulatory T cell recruitment and myeloid-derived suppressor cells [56,90]. Notably, CCL5 also acts as an auto-stimulatory signal for GBM cells by binding to the non-conventional receptor CD44, resulting in increased cell survival, invasion, and proliferation [91]. Therefore, targeting this zone of recurrence and immune exhaustion protected by intact barrier is key to enhancing the efficacy of immunotherapy. However, achieving this requires CTLs to traffic through the BBB.

The BBB is a highly regulated physical and metabolic barrier which extends from the CNS microvasculature to the endothelial cells of postcapillary venules [92]. During neuro-inflammation the permeability of the endothelial cells changes to allow for the entrance of lymphocytes into the CNS. This is achieved by changes in BBB junctional morphology that allow lymphocytes access either by squeezing between endothelial cells (paracellular diapedesis) or crossing through pores in the endothelial cell membrane (transcellular diapedesis) [93]. Recent single-cell RNA sequencing of the neuro-vasculature also shows enhanced endothelial cell expression of MHC class II genes in the disease state. The endothelial cell signature also changes from CNS specific to mirroring the periphery, thereby promoting immune trafficking from the blood (preprint [94]). The endothelial cells of the BBB are sealed by adherens junctions, a continuous series of complex tight junctions, and recently discovered tricellular junctions [95]. In the inflammatory state, these tricellular junctions have been suggested to be the primary site of cellular migration, through the downregulation of proteins (tricellulin and angulins) which normally maintain their morphology [95,96]. Interestingly, recombinant CCL2 and CCL5 administration was demonstrated in vitro to enhance T cell diapedesis through tricellular junctions. This may therefore offer a therapeutic strategy specifically to enhance paracellular crossing at the BBB, although their effect on recruiting regulatory T cells must also be considered [95,96].

Tight junctions can also be targeted to allow for entry of therapeutic agents [97,98]. These junctions are maintained on the basolateral side by the transmembrane adhesion proteins VE-cadherin and platelet endothelial cell adhesion molecule (PECAM)-1 [99,100]. The apical side of endothelial tight junctions is secured by occludin and claudin-1/3/5/12 [101]. Together, these proteins seal the tight junctions together by binding with each other on opposite endothelial cells, reducing the intercellular distance [101]. Claudin-5 is the most commonly expressed protein in tight junctions [101], and can be targeted with recombinant protein inhibitors such as the non-toxic C-terminal domain of the *Clostridium Perfringens* enterotoxin [102]. This can reversibly open endothelial tight junctions and allow ingress of therapeutic agents. Targeting of claudin-5 in vitro results in reduced paracellular diapedesis of lymphocytes while increasing transcellular diapedesis [103]. Other studies have also shown that knockout of adhesion molecules such as PECAM-1 does not result in enhanced paracellular movement, but instead increases migration via cell membrane channels [104]. Taken together, it becomes apparent that functional tight junction regulating proteins are required for paracellular diapedesis, and that disruption of these proteins may shift trafficking towards endocytic lymphocyte migration patterns similar to those found in neuroinflammatory CNS states [93].

The process of transcellular diapedesis is mediated by endocytosis at the endothelial cell membrane. This endocytosis occurs through vesicles containing caveolin (Cav)-1, which are increased in number during disease states such as EAE [105,106]. Regions of the BBB rich in Cav1 upregulate expression of adhesion receptors such as ICAM-1, capturing T cells at regions of the BBB where endocytic vesicles are present [107]. Interestingly, in inflammatory conditions such as EAE, ICAM-1 is highly expressed on the endothelium, and this over-expression promotes transcellular diapedesis. This contrasts with the resting state where low/intermediate expression of ICAM-1 favors paracellular diapedesis [108]. Therefore, promoting the expression of LFA-1 on T cells which can bind to over-expressed ICAM-1 may enhance T cell trafficking (therapeutic approaches described in the previous section). However, whether this effect also extends to CD8^+^ T cells in the context of glioblastoma is unclear.

Differential trafficking of T cell subsets was also demonstrated by experiments using Cav1^−/−^ mice which induced almost total loss of Th1 transcellular migration but did not impair migration of Th17 cells [109]. In EAE, Th17 T cells have been demonstrated to use CCR6 to bind CCL20 produced by the choroid plexus epithelial cells to gain access to the ventricular CSF [110,111]. When considering the CCR6–CCL20 axis for therapeutic targeting in glioblastoma, CD8^+^CCR6^+^ T cells also migrate towards CCL20 and blockade of CCL20 or CCR6 has also been demonstrated to reduce neuroinflammation in murine models of subarachnoid hemorrhage [112,113]. However, over-expression of CCL20 by tumors also correlates with tumor progression in multiple cancer types, as well as decreased survival [114]. Importantly though, the tumor-promoting effects of CCR6 signaling appear to rely on CCR6^+^ stromal cells but not CCR6^+^ immune cells [114]. Upregulation of CCR6 on immune cells may therefore be the more prudent therapeutic approach for enhancing T cell infiltration while maintaining tumor control. Transforming growth factor (TGF)-β has been shown to promote CCR6 expression on human CD4 T cells but is also implicated in the promotion of regulatory FOXP3 expression [115]. However, TGF-β priming also generates a fractional population of CCR6^+^FOXP3^−^ cells [116]. Further selection of this population would therefore be desirable to achieve a pro-infiltrative, effector T cell phenotype. Models of EAE have also found that increased expression of CCL19 and CCL21 from mononuclear inflammatory cells binds CCR7^+^ T cells in the CSF [117]. CCL19 has been shown to enhance the frequency of antigen responsive IFN-γ^+^ CD8^+^ T cells in viral infection and CCR7 chemotaxis may be stimulated in vitro using by-products of coagulation factor XIIa (high-molecular-weight kininogen domain 5) [118,119]. However, CCL19 may also promote the migration of regulatory T cells (CD4^+^CD25^+^FoxP3^+^) and therefore its usefulness in glioblastoma is unclear [120].

While these mechanisms are of interest therapeutically to allow T cells to cross the BBB from the periphery, this is only the initial step in accessing the parenchyma. Interaction with professional antigen-presenting cells in the perivascular spaces is a key step before penetration of the glia limitans, which lines the blood vessels and the surface of the brain [121].

## 4. The Glia Limitans—Accessing the Parenchyma

Between the outer BBB and the parenchyma lies the glia limitans. The glia limitans is formed by astrocyte foot processes associating with the basal lamina of the parenchyma [110]. It is divided into two membranes: the glia limitans perivascularis (surrounding blood vessels) and the glia limitans superficialis (covering the surface of the brain) [122]. In much of the brain, these two membranes lie so closely together that they are indistinguishable, but beyond the capillaries at the venules, inflammation can cause these two membranes to separate, forming a perivascular space. This space communicates with the CSF and allows for APCs to present antigens to entering T cells [123]. This interaction is critical in allowing T cells to access the parenchyma—indeed, the effects of T cells in EAE only begin once immune cells have crossed the glia limitans [124]. The APC–T cell interaction drives the production of further pro-inflammatory cytokines which triggers the recruitment of more immune cells [111,125]. Interestingly, while the initial T cells that enter these perivascular spaces tend to have increased expression of CCR6, further recruitment occurs in a CCR6-independent manner [110,111]. This would suggest that CCR6^+^ T cells form part of an initial ‘licensing’ step and that their interaction with APCs in the perivascular spaces facilitates further entry of T cells in a non-CCR6-specific manner.

In normal physiology, T cell crossing at the glia limitans is mediated by the expression of laminins [126]. For example, the parenchymal membrane of the glia limitans contains α1 and α2 laminins [127], which CD4^+^ T cells are unable to bind in the non-inflammatory state. However, in EAE, CD4^+^ T cells can bypass this control mechanism by using matrix metalloproteinases (MMPs) which disrupt the astrocytic foot processes, breaking down barrier integrity and allowing for T cell ingress [124]. While this might suggest that MMP agonism may be an attractive prospect for opening the glia limitans, MMPs are involved in the angiogenesis and invasion of glioma [128]. Inhibition of MMP was even trialed using a broad-spectrum MMP inhibitor, but this resulted in widespread reports of musculoskeletal toxicity due to on-target, off-tumor effects [129,130]. Given these experiences, it is unlikely that MMP agonism in glioblastoma will be a desirable therapeutic target.

Another mediator of T cell entry into the parenchyma is CXCL12. In murine models, T cells have been noted to be held in perivascular spaces due to expression of CXCL12 [131]. This ‘hold’ is released in inflammatory conditions, as increased levels of IL-17 drive the expression of CXCR7 on endothelial cells, resulting in the internalization of CXCL12 [132]. This leads to increased CXCR4 expression on T cells and subsequent T cell entry into the parenchyma [131,132]. However, when considering the downregulation of CXCL12 as a therapeutic strategy, it is worth noting that recent studies evaluating T cell responses to viral infection in vitro have found that CXCL12 at the BBB endothelium can promote CD8^+^ migration across the BCSF interface, suggestive of a location-dependent role [133]. A summary of these selected targets and therapeutic considerations is shown in Table 1.

## 5. T Cell Trafficking through the Parenchyma

Once past the glia limitans, effector T cells must reach and infiltrate the tumor to exert their cytotoxic effect. As discussed in the introduction, glioblastoma can restrict T cell trafficking due to the downregulated expression of VEGF-C, resulting in restricted lymphangiogenesis [22]. Notably, in patients treated with neoadjuvant anti-PD-1, VEGF-C expression was highly correlated with increased infiltration of T cells [138]. Thus, restoring levels of lymphangiogenesis-promoting factors such as VEGF-C could also enhance T cell homing and infiltration to the tumor. This is supported by the findings of Song et al., who demonstrated that intra-cisterna magna injections of an adeno-associated viral vector coding for VEGF-C could remodel meningeal lymphatic vessels in murine models of glioma [22]. Further enhanced expression of VEGF-C in lymphatic endothelial cells could potentiate the effect of checkpoint blockade due to enhanced T cell infiltration [22].

**Figure 2:**
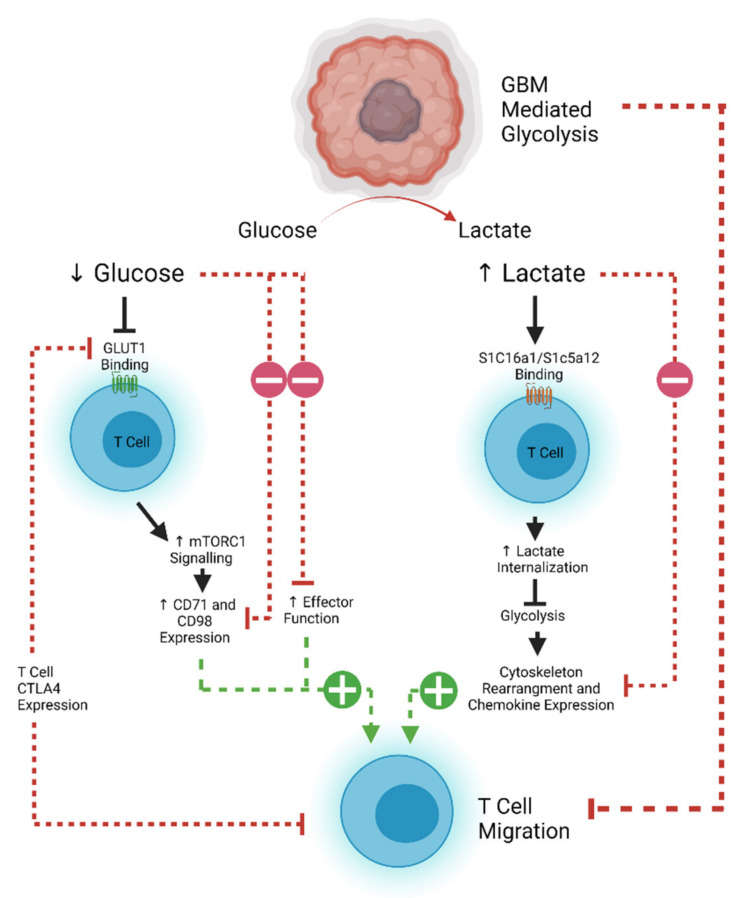
Glioblastoma effects on T cell metabolism and motility. As a rapidly dividing tumor, glioblastoma rapidly takes up glucose and produces lactate (the Warburg effect). Lack of glucose results in decreased GLUT1 binding (also downregulated by CTLA4) and downregulates effector function and motility. Increased lactate is internalized in T cells, where it also inhibits glycolysis and interferes with cytoskeleton rearrangement, resulting in decreased T cell migration. Produced using Biorender.

T cell motility is also dependent on metabolic pathways that are often usurped by rapidly proliferating tumors. Tumor cells demonstrate increased glucose uptake and lactose production, even in the presence of oxygen and functioning mitochondria (known as the Warburg effect) [139,140]. This affords the tumor and other rapidly proliferating cells essential anabolic precursors for cell proliferation [140]. The increased glucose demand by tumor cells therefore decreases the amount available for circulating T cells to maintain effector and migratory function [141]. Aerobic glycolysis is the main source of ATP production in leukocytes, which is required for the energetic demands of migration [142,143]. Inhibition of the T cell glycolytic pathway through administration of 2-DG and rapamycin causes a decrease in naïve T cell motility, demonstrating the importance of glucose in T cell homing [144,145]. The associated build-up of lactate caused by the Warburg effect also results in decreased migration of CD4^+^ T cells and a loss of cytolytic function of CD8^+^ T cells by interfering with T cell glycolysis [145,146,147,148]. However, this effect can be reversed, as demonstrated in an animal model of peritonitis where antibody-mediated blockade of lactate transporters on T cells allowed them to maintain their migratory potential [149]. Expression of CTLA-4 decreases the expression of the glucose transporter GLUT-1 on T cells, and further decreases effector function, implying that combinatorial approaches using checkpoint blockade may aid with T cell trafficking as well as reinvigoration of function [142,150]. However, recent work suggests that exhausted human CD8^+^ T cells may actually become more mobile [151]. CTLA-4 signaling can lead to a RAP1-mediated increase in LFA-1 binding, which can induce migration [152]. This has potential implications for considering which form of ICI would best work with a tumor where T cell trafficking poses a significant challenge. An overview of the metabolic pathways limiting T cell efficacy in glioblastoma is shown in Figure 2.

Another mediator of T cell glycolysis is the PI3K/AKT/mTOR pathway, whose activation can also downregulate the expression of adhesion and migration molecules CD62L, CCR7, and S1P1 in CD8^+^ T cells [142,153]. Loss of S1P1 has been shown to mediate T cell sequestration in bone marrow in glioblastoma, while S1P1^+^ cells are resistant to sequestration and can return into the circulation [142,154,155,156]. Therefore, reversing sequestration will be critical for future immunotherapy efficacy and is currently the subject of ongoing therapeutic investigation [157]. While one approach may be to inhibit the PI3K/AKT/mTOR pathway, this inhibition must be selective, as AKT possesses three isoforms which have varying pro- and anti-tumor effects. AKT signaling also plays an important role for the development of effector-like memory CD8^+^ T cells necessary for tumor immune surveillance [158]. Interestingly, recent work has described small-molecule inhibitors that may be capable of targeting pathogenic AKT isoforms only (AKT1 and AKT2) while leaving the tumor-suppressive functionality of AKT3 intact [159,160]. Indeed, specific AKT1 and 2 inhibition has been associated with enhanced central memory CD8^+^ T cell proliferation with prolonged cytokine and Granzyme B production, making this a potential future therapeutic strategy [158,159,160,161].

## 6. The Tumor Microenvironment

Once T cells traffic past the BBB and through the parenchyma, they will encounter the highly immunosuppressive tumor microenvironment. This is made up of regulatory T cells (CD4^+^CD25^+^FOXP3^+^), tumor-associated macrophages (TAMs) and myeloid-derived suppressor cells (MDSCs), as well as other stromal cells such as GSC-derived pericytes [23,162]. These can all work to suppress effector T cell function. Regulatory T cells induce T cell exhaustion and apoptosis, signaling via programmed death-ligand 1 (PD-L1), cytotoxic T-lymphocyte-associated protein 4 (CTLA-4), lymphocyte activation gene 3 (LAG-3), T cell immunoglobulin and mucin domain-containing protein 3 (TIM-3), and others [163,164]. They also can dampen the production of inflammatory cytokines and CTL proliferation by downregulating interleukin-2 and interferon-γ [165]. Gliomas are adept at recruiting regulatory T cells to the microenvironment by over-production of factors such as indoleamine 2,3 -dioxygenase-1 (IDO-1) [166]. As mentioned previously, GSC-derived pericytes also secrete CCL5, which can promote the recruitment of regulatory T cells to the TME [54]. Stromal cells in the microenvironment also produce highly immunosuppressive cytokines, such as transforming growth factor β (TGFβ) and interleukin-10 (IL-10) [167,168].

Despite the numerous targets for blockade, it is notable that ICI and the interruption of pro-tumor metabolic pathways have failed as a monotherapy [9,169]. Increasingly, attention is turning towards combinatorial therapies, where multiple drivers of T cell exhaustion can be blocked simultaneously [170]. This includes using bispecific antibodies against TGF-β and PD-L1 or against PD-L1 and the anti-agonist CD27 [171,172,173]. These approaches are currently being evaluated in Phase I trials in advanced solid tumors (NCT04429542, NCT04440943). Cytokine modulation approaches are also a potential avenue for enhancing T cell activity in the TME, as seen in ‘armored’ CAR-T constructs. The addition of IL-12, IL-15, or IL-18 along with antigen specificity to T cells appears to result in greater CTL activity and anti-tumor efficacy [174,175,176]. A high percentage of regulatory T cells in the peripheral blood of GBM patients express CCR4 compared to controls (74 vs. 43%) [135]. CCL4 binds CCL22 (and others), which has been shown to be overexpressed in freshly resected human glioma cells, and blockade of CCR4 in vitro can significantly reduce regulatory T cell migration [135]. Targeting fibroblast activation proteins or introducing heparinase-expressing agents may also help to disrupt immunosuppressive stromal elements [146,177,178]. Intratumoral APCs are also necessary to stimulate and retain infiltrating lymphocytes at the tumor site, as well as carrying antigens to draining lymph nodes and cross priming peripheral CD8 T cells [179,180,181]. The administration of intratumoral FMS-like tyrosine kinase 3 ligand (Flt3L) and poly I:C has been shown to expand and mature dendritic cell precursors, resulting in greater antitumor efficacy when combined with immunotherapies such as PD-L1 blockade or oncolytic herpes simplex viruses [179,182].

Standard-of-care therapies also can help drive a more potent immune response. Temozolomide (TMZ) is an alkylating chemotherapy whose main function is to induce DNA double-stranded breaks, resulting in tumor cell death [183]. Interestingly, TMZ can also help to reduce the numbers of peripheral regulatory T cells, as well as interrupting their migration [136,184]. In disease states such as glioblastoma, tumor cells and platelet-derived growth factor receptor beta (PDGFRβ)-expressing cells of the neurovascular sub-units (such as pericytes and perivascular fibroblast-like cells) produce CCL2 to recruit regulatory T cells and dampen the effector response [185]. TMZ interrupts the CCL2–CCR4 axis, thereby reducing this effect [136,184]. Combining immunotherapy with radiotherapy also can help to polarize the T cell response to a cytotoxic phenotype by inducing greater T cell receptor diversity and expanding the numbers of tumor-infiltrating lymphocytes and effector memory T cells [186]. In pre-clinical murine models of glioma, radiotherapy combined with antibodies against markers of exhaustion such as TIM-3 and PD-1 was able to produce long-term survival [11].

## 7. Modeling the BBB

Animal and in vitro models have contributed greatly to our knowledge regarding the cell and protein interactions required to cross the BBB. Rudimentary animal models from the 1980s first established how BBB permeability could change in response to systemic compounds by tracking the CNS uptake of Evan’s Blue dye following intravenous infusion [187,188,189,190]. These models established protocols to visualize membrane cellular components and tissue hierarchy through fluorescent microscopy and histology, allowing for the elucidation of fundamental mechanisms behind membrane permeability. Such models included mice with proteins essential for T cell chemotaxis across the BBB knocked out, including tight junction proteins claudin 5 [100] and occludin [191]. Unfortunately, full knockout of these proteins results in non-viable pups or other dysfunctional phenotypes, suggesting the importance of tight junction proteins in development. Additional knockout mice focusing on proteins involved in T cell rolling, p-selectin, and its ligand PSGL-1 [192] were developed and confirmed less BBB breakdown and leukocyte trafficking into the CNS. Similarly, the use of antibody natalizumab to block the α4 subunit on T cells has been successful in preventing BBB chemotaxis in MS [193]. Genetic models targeting pericyte and astrocyte function have also been generated to establish how these cell types support BBB formation in development and regulate tight junctions in injury and disease. The use of two-photon microscopy has allowed for imaging at depths up to 1mm, but real-time high-resolution imaging and cell tracking capabilities are limited. Animal models closely mimic BBB features by including all cell types within the vascular interface, fluid flow and biochemical concentrations. However, there are still challenges translating in vivo finding to clinical significance. Genetic, molecular, and immunological differences between humans and rodents, as well as high cost and ethical concerns with animal testing, have generated a need for robust in vitro models.

In vitro models of the BBB range from simple endothelial cell monolayers to complex three-dimensional systems with fluid flow and ionic gradients [194,195]. These models have the advantage of using human cells as well as being cost effective and allowing for high-throughput screening of a variety of different conditions or molecules. Transwell, hydrogels, and microfluidic devices with three or four different cell types have been created in attempts to best mimic native BBB function. Simplified in vitro models allow for researchers to specifically modify or track elements of the BBB. Cell types used in these models have traditionally been primary brain endothelial cells or immortalized cell lines. Immune factors affecting BBB permeability have been most studied with BECs due to their accurate expression of chemokine and cytokine receptors. Interestingly, these models found CCL2 to cause redistribution of tight junction proteins, such as claudin-5 and occluding, under physiological and pathological conditions [196,197], which gives insight into the mechanism of increased T cell chemotaxis during inflammation and elevated CCL2. Brain-cancer-specific models have focused on integrating vasculature and tumor cells to test the toxicity of therapeutics prior to animal studies [198,199]. These models recapitulated the three-dimensional structure of a brain tumor but used lung fibroblasts, HUVECs, and gelatin, which may not accurately represent the blood–brain barrier and brain microenvironment. The development of induced pluripotent stem cells (iPSCs) has allowed for genetically identical personalized in vitro models to test drug and cell interactions with BBB of specific individuals and disease conditions [200]. Overall, both in vivo and in vitro models of the BBB have limitations but can provide valuable insight to improve T cell chemotaxis in GBM.

## 8. Safety

While this review has largely focused on strategies by which T cells can be recruited and restored to a cytotoxic effector status, it must be noted that rapid increases in activated T cells in the circulation can potentially lead to cytokine release syndrome (CRS), mediated by the release of pro-inflammatory cytokines such as IL-6 [201]. Therefore, when considering therapies that will increase circulating activated T cells and subsequent CNS T cell infiltration, careful consideration must be given to the safety of any such approach. Such therapies may lead to systemic and neurological complications, even when not used specifically to treat CNS malignancies.

This is demonstrated by the example of clinically used therapeutics such as ipilimumab, which re-invigorates T cells by blockade of CTLA-4 [202]. Ipilimumab has been associated with pituitary inflammation (hypophysitis), occurring in up to 17% of patients receiving ipilimumab treatment [203,204]. Similar syndromes are also observed when using anti-PD-1 and anti-PD-L1 therapies, albeit at a lower frequency compared to anti-CTLA-4 [205]. The mechanism of how ipilimumab causes hypophysitis remains unclear, but it is speculated that ipilimumab can release the brakes on T cells that target and destroy pituitary cells, or that expression of CTLA-4 on pituitary cells leads to complement fixation mediated by ipilimumab, resulting in the destruction of pituitary cells [206,207]. In rarer circumstances (less than 0.2% of patients), ICI, especially ipilimumab or combination ipilimumab/nivolumab (anti-PD-1), has caused aseptic meningitis and encephalitis [208,209,210,211,212]. Like with hypophysitis, the exact mechanism is unclear. However, in the case of encephalitis, there is evidence that the effect is autoimmune in origin, as some patients treated with ICI exhibit autoantibodies to the NMDA receptor, a characteristic of other autoimmune encephalopathies [213,214]. ICI has also reportedly induced new CNS demyelination and exacerbated existing CNS demyelination in MS patients [215,216]. These rare, but serious neurological deficits resulting from systemic ICI emphasize the need for careful monitoring of patients receiving therapies that enhance T cell trafficking and function.

The experience of treatments using adoptively transferred chimeric antigen receptor (CAR) T cells in extracranial and intracranial malignancies can also be illustrative for potential systemic and neurological toxicities. The most common CAR-related toxicity is cytokine release syndrome (CRS), occurring in up to 37–93% of patients with lymphoma or leukemia receiving CD19 CARs [217]. As described previously, CRS is caused by rapid activation of CAR T cells upon administration and subsequent release of pro-inflammatory cytokines, such as IL-6 [201]. High levels of serum IL-6 were found to correlate with severe CRS, which led to the FDA approval of tocilizumab, an anti-IL-6 receptor antagonist [218]. Strategies to reduce CRS include administering lower doses of CAR T cells over multiple infusions as opposed to one single bolus [219,220].

Neurological-specific toxicities after CAR administration are also possible. Immune effector cell-associated neurotoxicity syndrome (ICANS) can develop in around 50% of patients following systemic CAR infusion [221]. ICANS manifests with minor symptoms such as lethargy and confusion but can also cause seizures and coma. The pathophysiology of ICANS remains unclear, but evidence suggests that release of pro-inflammatory cytokines, such as IL-6 and IL-1β, by CAR T cells can disrupt the BBB, resulting in the accumulation of CAR T cells and pro-inflammatory cytokines in the CNS [222]. Klinger et al. recently described a mechanism whereby CD19 bi-specific T cell engagers (blinatumomab) can induce T cell adhesion to endothelial cells of the BBB followed by T cell migration into the perivascular space in a CD19-independent manner. Once past the BBB, they may encounter rare target CD19 cells in the CNS and release pro-inflammatory cytokines, triggering ICANS-like symptoms [223]. However, unlike CRS, ICANS does not respond to tocilizumab treatment, and symptoms are typically managed with corticosteroids or cessation of therapy [224]. Klinger et al. also reported that the non-specific entry of CD19 T cells into the CNS could be abrogated by the administration of anti-adhesion agents (anti-VLA4, natalizumab), offering another potential therapeutic if toxicity occurs [223]. In summary, while enhanced T cell chemotaxis and infiltration of glioblastoma are necessary for effective immune-mediated treatment of tumors, this must be carefully balanced with the risks described above.

## 9. Conclusions

For immunotherapy in glioblastoma to be successful, sustained recruitment of effector lymphocytes from the periphery to the tumor is necessary. However, achieving this in a unique immune environment such as the CNS must overcome both physical and chemical barriers. In this review, we have described the process by which effector T cells can be recruited from the periphery and what modifications may result in a pro-infiltrative phenotype. We have described both T cell and BBB factors that would be desirable therapeutic targets and set out strategies by which this may be achieved. Adhesional factors on the BBB endothelium such as ICAM-1, VCAM-1, or ACKR1 may be upregulated by IL-1β or IFN-γ, which can be delivered via convection-enhanced delivery (CED) directly to the tumor site. Delivery of these cytokines and other inflammatory factors can have profound effects on increasing BBB penetration and the migration potential of T cells. Induced expression of CXCL10 by using poly-ICLC can also interact with CXCR3 on effector T cells, prompting their infiltration into tumor. Co-culture with IL-12 may help drive the expression of key integrins such as LFA-1 on the surface of T cells in preparation for adoptive transfer to further enhance their adhesive capabilities. CCL2 and CCL5 may promote paracellular diapedesis through tricellular junctions in the BBB endothelium, while TGF-β priming of T cells can increase their CCR6 expression, which can promote transcellular crossing. However, CCL2 and CCL5 may also mediate regulatory T cell recruitment, perhaps necessitating co-administration with checkpoint blockade. Subsequent navigation through the glia limitans may be aided by IL-17-mediated downregulation of CXCL12, although this may be a location-specific effect. Once inside the parenchyma, lymphangiogenesis-promoting factors such as VEGF-C may further enhance trafficking of T cells to the tumor. Metabolic mediators such as the PI3K/AKT/mTOR pathway may also be therapeutically targeted using small-molecule inhibitors of the AKT1 and AKT2 isoforms. Combinatorial approaches to stimulate T cells and block checkpoint inhibition will likely be necessary to overcome the microenvironment. This may be achieved using novel bispecific constructs or co-administration with immune stimulatory cytokines such as IL-12, IL-15, or IL-18. Standard-of-care therapies such as TMZ and radiotherapy may also help to blockade regulatory T cell recruitment and drive a more diverse and potent T cell response. Importantly, however, any approach that enhances T cell infiltration into the CNS must consider safety, and although there are therapeutic options for adverse events, future trial designs using pro-infiltrative therapies should err on the side of caution. Nevertheless, enhanced T cell trafficking and infiltration of glioblastoma is essential for immunotherapeutic efficacy. While ICI seeks to ‘release the brakes’ on T cell activity, in the case of glioblastoma, we must first drive T cells to the tumor.

## Figures and Tables

**Table 1:** A summary of selected factors that may enhance trafficking and infiltration of T cells across the BBB.

Interactor	Behavior	Therapeutic Considerations	References
T cell processes			
LFA-1	T cell integrin which binds ICAM-1. Promotes T cell capture and rolling in inflammatory and non-inflammatory state.	IL-12 induces LFA-1 expression and can enhance T cell migration in several murine malignancies.	[36,73]
VLA-4 (α4β1)	Integrin on T cell which binds VCAM-1 in the inflammatory state and interacts with other transmembrane proteins (JAM-B, JAML, etc.).	IL-12 induces LFA-1 and VLA-4 expression and enhances T cell migration in several murine malignancies. Effect may be malignancy dependent.	[41,42,73]
	CXCL9: Polarizes T cells to a Th1/Th17 phenotype.	Mediated lymphocyte infiltration and suppresses tumor growth in cutaneous fibrosarcoma.	[134]
CXCR3 (3 ligands)	CXCL10: Only moderately induces CXCR3 internalization and enhances T cell infiltration.	DPP-4 blockade increases TILs but is also tumorigenic (independent of enzymatic function). Combinatorial poly-ICLC enhances CXCL10 expression.	[81,82,83]
	CXCL11: Binds CXCR3 strongly and induces receptor internalization.	Promotes lineage of regulatory T cells.	[75]
CCR4	CCL2, CCL22 (and others): Overexpressed on glioma cells, recruits regulatory T cells.	CCR4-CCL22 signaling recruits regulatory T cells. Blockade of CCR4 in vitro can reduce regulatory T cell migration. TMZ can also mitigate production of CCL2.	[135,136]
CCR5	Binds CCL3, CCL4, and CCL5. May help to recruit cytolytic T cells but also regulatory T cells.	CCL4 can help recruit cytolytic CCR5^+^ T cells in esophageal squamous cell carcinoma but CCL4–CCR5 interaction can enhance the invasion ability of glioblastoma in vitro. CCL5 is also associated with enhanced T cell diapedesis at tricellular junctions. However, CCL5 also binds CD44 on GBM cells to drive proliferation and survival and is produced by perivascular stromal cells such as pericytes. Blockade of CCR5 (maraviroc) may limit cancer-associated fibroblast accumulation.	[54,62,63,91,95]
CCR6	Binds CCL20 expressed at the choroid plexus. CD8^+^ T cells migrate to CCL20 in murine SAH.	TGF- β promotes CCR6 expression but also is implicated in the promotion of FOXP3^+^ cells. However, a fraction of the population is CCR6^+^FOXP3^−^. CCR6 T cells may also be involved with licensing further recruitment to perivascular spaces.	[115,116]
CCR7	Present on activated CD8 T cells (and central memory T cells).	Interacts with CCL19 and may mediate integrin activation on immune cells or diapedesis. Chemotaxis may be enhanced by a peptide derived from the byproduct of coagulation factor XIIa cleavage. May also promote regulatory T cells.	[117,119,120]
**Blood–brain barrier processes**			
E/P-Selectin	Expressed in inflammatory state only. Binds PSGL-1^+^ CD8 T cells, slowing them on BBB endothelium.	Expression enhanced in response to inflammatory cytokines (e.g., IL-1 or TNF α). IL-1 has been delivered via CED in rat models of glioma.	[45,137]
Claudin-5, PECAM-1	Commonly expressed proteins involved in sealing tight junctions at BBB.	Modified Clostridium perfringens enterotoxin can reversibly open tight junctions. May drive T cells to transcellular migration.	[93,103,104]
ACKR1	Trafficking of pro-infiltrative chemokines from abluminal to luminal surface of BBB.	IL-1 signaling associated with upregulated expression ACKR1 (along with VCAM-1, ICAM-1). Trialed using CED in rat glioma.	[45]
Caveolin-1	Expressed in endocytic vesicles at BBB and acts as a mediator of transcellular diapedesis.	Regions of BBB rich in CAV-1 are also rich in ICAM-1. Enhancing ICAM-1 on BBB (e.g., via IL-1) may capture more T cells that can undergo para and transcellular diapedesis.	[108]
CXCL12	Acts as a T cell, holding factor cells in perivascular spaces. Expression of CXCR7 on endothelial cells internalizes CXCL12.	IL-17 drives expression of CXCR7 on endothelial cells and CXCR4 on T cells which licenses their entry into the parenchyma. However, CXCL12 may promote CD8^+^ migration across BCSF barrier—may be a location-specific role.	[131,132,133]

This table only provides selected examples and is not exhaustive.
